# Tocopheryl Phosphate Inhibits Rheumatoid Arthritis-Related Gene Expression In Vitro and Ameliorates Arthritic Symptoms in Mice

**DOI:** 10.3390/molecules27041425

**Published:** 2022-02-20

**Authors:** Susumu Hama, Naoko Kirimura, Aki Obara, Hirokatsu Takatsu, Kentaro Kogure

**Affiliations:** 1 Faculty of Pharmacy, Research Institute of Pharmaceutical Sciences, Musashino University, Tokyo 202-8585, Japan; s-hama@musashino-u.ac.jp; 2 Department of Biophysical Chemistry, Kyoto Pharmaceutical University, Kyoto 607-8414, Japan; naoko_musochi@icloud.com; 3 Department of Analytical Chemistry, Kyorin University, Tokyo 181-8612, Japan; obara@ks.kyorin-u.ac.jp (A.O.); takatsu@ks.kyorin-u.ac.jp (H.T.); 4 Graduate School of Biomedical Sciences, Tokushima University, Tokushima 770-8505, Japan

**Keywords:** α-tocopherol, tocopheryl phosphate, tocopheryl succinate, rheumatoid arthritis

## Abstract

Anti-rheumatoid arthritis (RA) effects of α-tocopherol (α-T) have been shown in human patients in a double-blind trial. However, the effects of α-T and its derivatives on fibroblast-like synoviocytes (FLS) during the pathogenesis of RA remain unclear. In the present study, we compared the expression levels of genes related to RA progression in FLS treated with α-T, succinic ester of α-T (TS), and phosphate ester of α-T (TP), as determined via RT-PCR. The mRNA levels of interleukin (IL)-6, tumor necrosis factor-α (TNF-α), matrix metalloproteinase (MMP)-3, and MMP-13 were reduced by treatment with TP without cytotoxicity, while α-T and TS did not show such effects. Furthermore, intraperitoneal injection of TP ameliorated the edema of the foot and joint and improved the arthritis score in laminarin-induced RA model mice. Therefore, TP exerted anti-RA effects through by inhibiting RA-related gene expression.

## 1. Introduction

Rheumatoid arthritis (RA) is an autoimmune disease characterized by pannus formation involving activated fibroblast-like synoviocytes (FLS) [1,2]. Pannus, a thick and swollen synovial membrane, consists of myofibroblast, fibroblast and inflammatory cells [1]. Intercellular communication between FLS and immunocompetent cells through inflammatory cytokines is important for pannus formation [1,2,3]. Within the joints of RA, synovial cells proliferate to form pannus, and immunocompetent cells enter the synovial tissue via neovascularization, followed by immune activation, which leads to pain, arthritis, and joint swelling [1,2]. Pannus in the vicinity of the bone also causes joint destruction by secreting large amounts of matrix metalloproteinase (MMP) [1,2]. Thus, activated FLS, which play a central role in the pannus, contribute to the pathogenesis of RA.

Tumor necrosis factor (TNF)-α is an important proinflammatory cytokine responsible for pannus formation, impacting FLS activity [4,5]; it induces FLS proliferation, vascular endothelial cell activation, expression of the proinflammatory cytokine IL-6, and MMP expression [6,7,8,9]. Thus, TNF-α-stimulated FLS have been used to analyze the pathogenesis of RA [10,11,12,13]. IL-6 also induces autoantibody production, vascular endothelial growth factor expression, T cell activation, and osteoclast differentiation [14,15,16,17]. Thus, TNF-α and IL-6 are major proinflammatory cytokines that are pivotally involved in the pathogenesis of RA, and TNF-α and IL-6 inhibitors are used clinically to treat RA [18,19,20,21]. MMP-3 and MMP-13, which are induced by TNF-α [9], promote joint destruction by degrading collagen and fibronectin in cartilage tissue.

Previous studies have investigated the effect of antioxidants on FLS because reactive oxygen species induce inflammatory cytokine expression [22,23,24]. α-Tocopherol (α-T) is a lipophilic antioxidant and one of the most bioactive forms of vitamin E [25,26]. Previously, the efficacy of oral administration of α-T in rheumatic patients was evaluated in a placebo-controlled double-blind trial [27]; α-T was shown to have analgesic activity without inflammatory activity or oxidative modification. Therefore, there are many unclear points regarding the antirheumatic effects of α-T. In particular, the effect of α-T on FLS, which is pivotal in the pathogenesis of RA, has not been fully investigated. In the present study, we used FLS to investigate the effect of α-T on the expression of the RA-related genes TNF-α, IL-6, MMP-3, and MMP-13. In addition, we examined the effects of α-T derivatives, specifically α-tocopheryl succinate (TS) and α-tocopheryl phosphate (TP), on FLS. TS is a succinate ester of α-T that not only induces cancer cell-specific apoptosis and inhibits angiogenesis, but also inhibits the transcriptional activity of TNF-α [28,29,30]. TP is a phosphate ester of α-T and is water-soluble and chemically stable. TP reportedly exhibits various cellular regulatory effects of TP, including inhibition of CD36 scavenger receptor surface exposure, oxidized low-density lipoprotein uptake, stimulation of the PI3K signaling pathway and angiogenesis in cultured cells [31,32,33,34,35]. Furthermore, TP interacts with the cannabinoid receptor system, protects against ultraviolet-induced skin damage, prevents atherosclerosis, and has anti-inflammatory effects through the reduced presentation of CD36 to the cell surface and diminished expression of inflammatory cytokines IL-6 and TNF-α in animal studies [31,36,37,38,39].

In the present study, we examined the effects of α-T, TS, and TP on the expression of RA-related genes in FLS, and determined the in vivo anti-RA effects of TP on laminarin-induced RA model mice.

## 2. Results and Discussion

### 2.1. Effects of α-T, TS, and TP on the Viability of FLS

**Figure 1:**
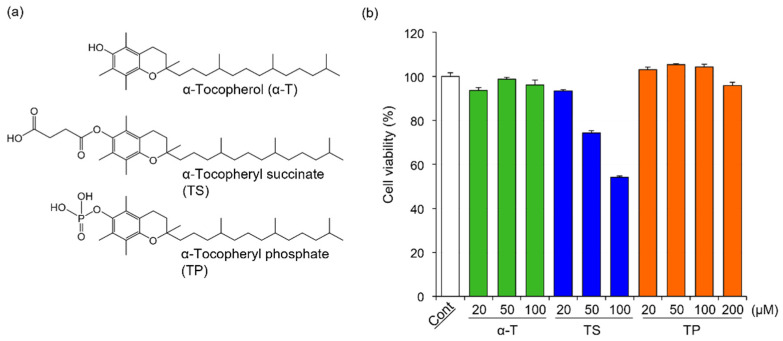
Chemical structures of α-T, TS, and TP, and their effects on the viability of HFLS-RA. (**a**) Chemical structures of α-T, TS, and TP, (**b**) Effects of α-T, TS, and TP on the viability of HFLS-RA. HFLS-RA cells were treated with 50 μM α-T, 20 μM TS and 50 μM TP for 24 h. The cell viability was determined by WST-1 assay. Values represent the means of three individual experiments. Bars represent SD.

The chemical structures of α-T, TS, and TP are shown in Figure 1a. In general, cell viability affects the expression of various genes. To determine the concentration of α-T, TS, and TP without cytotoxicity in FLS, we investigated the effects of α-T, TS, and TP on the viability of FLS. The viabilities of FLS treated with α-T and TP were comparable to those of untreated cells (Figure 1b). In TP-treated FLS, the viability did not change even when the cells were treated with concentrations greater than 100 μM. On the other hand, the viability of FLS was inhibited by treatment with TS at concentrations greater than 50 μM (Figure 1b). These results suggested that α-T and TP showed no cytotoxicity against FLS at the indicated concentrations, whereas TS was cytotoxic at concentrations over 50 μM. It has been reported that reactive oxygen species (ROS) induce apoptosis in FLS [40,41]. In various cell lines, TS induces apoptosis through the production of superoxide anion radicals [28,42]. Although α-T has no cytotoxicity in various cells because it is a major antioxidant, it has been reported that the cytotoxicity of TP depends on the concentration of treatment [32,43,44]. At low concentrations, TP protects against ROS-mediated cell death through the inhibition of peroxides, lipid hydroperoxides, and superoxide anion radicals such as α-T [44]. Therefore, FLS might show high sensitivity to ROS, with TS (≥50 μM) showing cytotoxicity in FLS, whereas α-T and TP showed no effects.

In this study, to determine the effects of α-T, TS, and TP on rheumatoid arthritis-related gene expression without cytotoxicity, the concentrations of α-T, TS, and TP were set at 50, 20, and 50 μM, respectively.

### 2.2. Effects of α-T, TS, and TP on the Expression of Rheumatoid Arthritis-Related Genes

**Figure 2:**
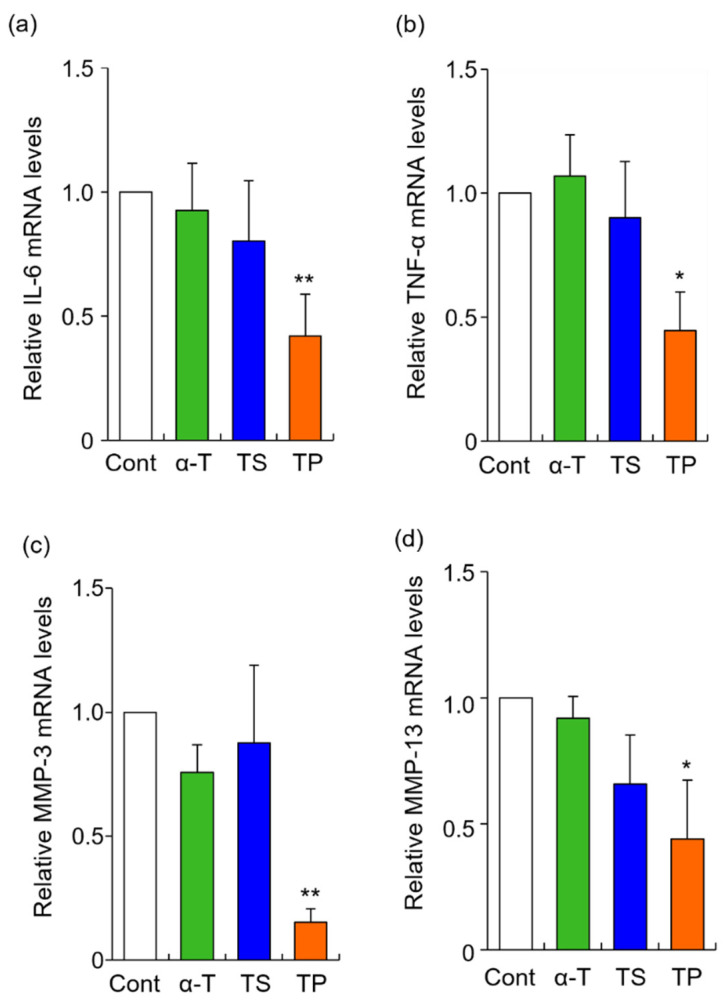
Effects of α-T, TS, and TP on the expression of IL-6, TNF-α, MMP-3 and MMP-13 mRNA in HFLS-RA. HFLS-RA cells were treated with 50 μM α-T, 20 μM TS, and 50 μM TP for 24 h. IL-6 (**a**), TNF-α (**b**), MMP-3 (**c**), and MMP-13 (**d**) mRNA levels were determined by RT-PCR with these specific primer sets. Values represent the means of three individual experiments. Bars represent SD. * *p* < 0.05 and ** *p* < 0.01.

The rheumatoid arthritis-related genes, IL-6, TNF-α, MMP-3, and MMP13, were selected in this study. The mRNA levels of IL-6, TNF-α, MMP-3, and MMP13 were significantly inhibited by TP treatment alone, whereas α-T did not alter the expression of any gene examined in the present study (Figure 2). To determine whether the site of action of TP affects gene expression, we quantified the amount of TP in the culture medium by HPLC-FL. When FLS were treated with 50 μM TP for 24 h, the concentration of TP was 33.3 μM. When the culture medium containing 50 μM TP was incubated without FLS for 24 h, the concentration of TP was 35.2 μM. The 14.8 μM decrease in concentration when TP was incubated without FLS might be due to hydrolysis of TP or non-specific adsorption to the culture plate. Based on these results, the cellular uptake efficiency of TP was estimated as follows: cellular uptake efficiency = {(TP concentration without FLS: 35.2 μM) -− (TP concentration with FLS: 33.3 μM)}/(applied dose: 50 μM) × 100. Thus, 3.8% of the applied dose of TP was taken up by the FLS. This result suggested that TP molecules were taken up by FLS, inducing the inhibitory effects of IL-6, TNF-α, MMP-3, and MMP-13. It has previously been reported that the cellular uptake of TP is mediated by organic anion transporters in the human monocytic leukemia cell line THP-1 [45]. Furthermore, CD36 and SR-BI scavenger receptors are involved in the cellular uptake of TP in THP-1 cells [35]. However, organic anion transporters, CD36 and SR-BI scavenger receptors might not necessarily participate in the cellular uptake of TP in HFLS-RA cells, as the expression and activity of these proteins might differ between THP-1 and HFLS-RA cells.

In the present study, α-T did not inhibit the expression of RA-related genes. However, it has previously been reported that TP is hydrolyzed within cells and converted to α-T in vivo [46]. Although further investigation is needed, TP may act as a provitamin of α-T.

**Figure 3:**
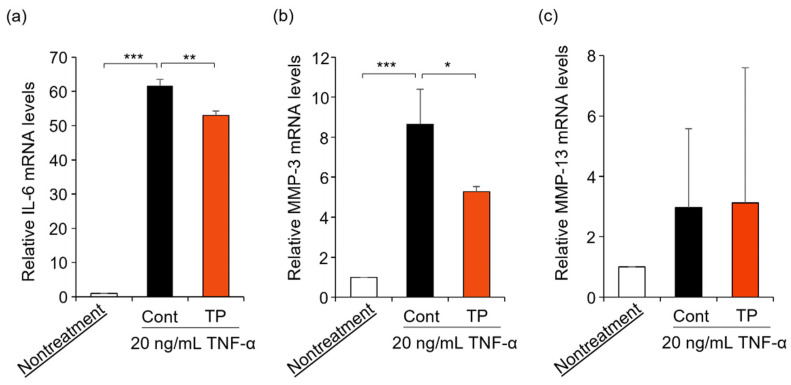
Effects of TP on the expression of IL-6, MMP-3 and MMP-13 mRNA in HFLS-RA cells stimulated with TNF-α. HFLS-RA cells were treated with 50 μM TP in the presence of 20 ng/mL TNF-α for 24 h. IL-6 (**a**), MMP-3 (**b**) and MMP-13 (**c**) mRNA levels were determined by RT-PCR with specific primer sets. Values represent the means of three individual experiments. Bars represent SD. * *p* < 0.05, ** *p* < 0.01 and *** *p* < 0.001.

FLS are key effector cells for understanding the pathology of RA, with FLS being activated by TNF-α treatment [4,5,6,7,8,9]. Furthermore, TNF-α activates the gene expression of IL-6, MMP-3, and MMP-13 [9]. Therefore, we examined the effects of TP on RA-related mRNA expressions in TNF-α-stimulated FLS. As shown in Figure 3, TP treatment significantly suppressed IL-6 and MMP-3 mRNA levels, which were upregulated by TNF-α, while MMP-13 mRNA levels remained unaltered. These results suggest that TP decreased IL-6 and MMP-3 mRNA levels by inhibiting TNF-α signaling, although additional investigations are warranted.

### 2.3. Anti-RA Effects of TP on Laminarin-Induced RA Model Mice

**Figure 4:**
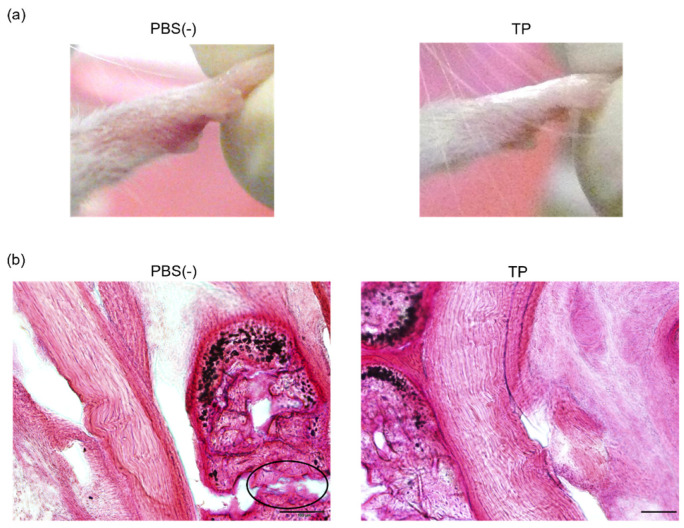
Anti RA effects of TP in laminarin-induced RA model mice. PBS (−) and TP dissolved in PBS (−) were intraperitoneally administered into mice thrice weekly, 7 days after laminarin injection. (**a**) Typical images of forefoot 21 days after laminarin injection. (**b**) Hematoxylin and eosin stained histological images of joint of forefoot at 49 days after laminarin injection. Area surrounded by a circle of black line indicates infiltrated inflammatory cells.

**Figure 5:**
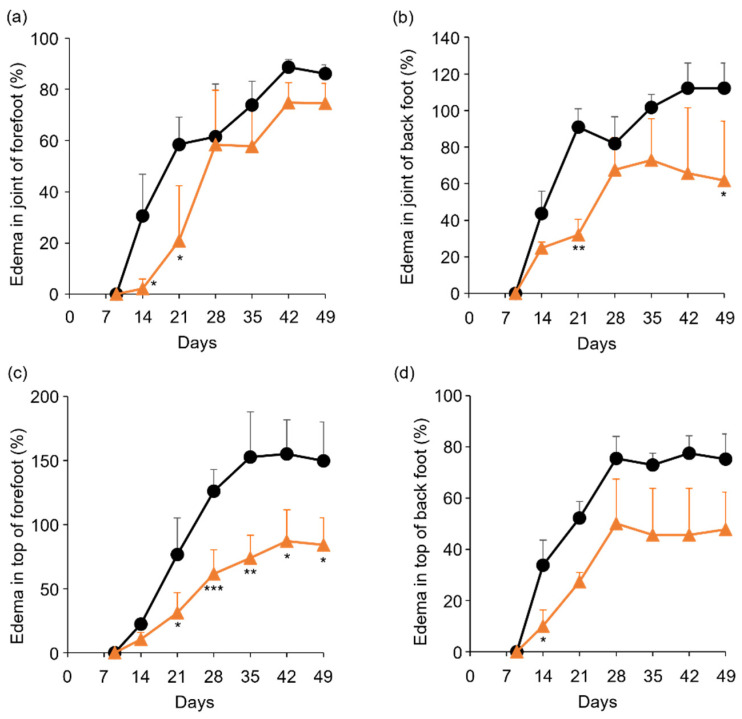
Comparison of edema ratio (%) between control and TP-administered mice. (**a**–**d**) PBS (−) (black lines) and TP dissolved in PBS (−) (orange lines) were intraperitoneally administered into mice three times a week from 7 days after laminarin injection. Edema ratios were calculated as described in materials and methods section. (**a**,**b**) Joint of forefoot (**a**) and back foot (**b**). (**c**,**d**) Top of forefoot (**c**) and back foot (**d**). *N* = 3, Bars represent SD. * *p* < 0.05, ** *p* < 0.01 and *** *p* < 0.001.

**Figure 6:**
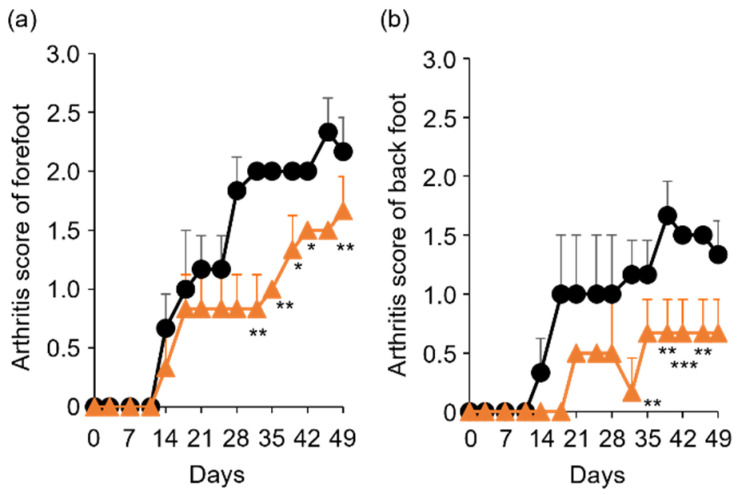
Comparison of arthritis score between control and TP-administered mice. (**a**,**b**) PBS (−) (black lines) and TP dissolved in PBS (−) (orange lines) were intraperitoneally administered into mice three times a week from 7 days after laminarin injection. Arthritis scores of forefoot (**a**) and back foot (**b**) were evaluated as described in materials and methods section. *N* = 3, Bars represent SD. * *p* < 0.05, ** *p* < 0.01 and *** *p* < 0.001.

**Figure 7:**
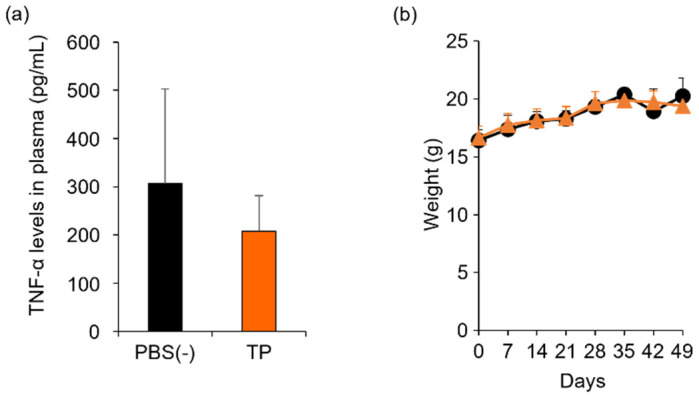
Comparison of plasma TNF-α levels and body weight between control and TP-administered mice. PBS (−) (black) and TP dissolved in PBS (−) (orange) were intraperitoneally administered into mice thrice weekly, 7 days after laminarin injection. (**a**) Plasma TNF-α levels at 26 days after laminarin injection. (**b**) Body weight. *N* = 3, Bars represent standard deviation (SD).

To determine the in vivo anti-RA effects of TP, especially arthritis, TP was intraperitoneally administered to laminarin-induced RA model mice. As shown in Figure 4a, laminarin-induced swelling, reddening, and edema were inhibited by TP treatment in comparison with the control group and TP-administered mice 21 days after the laminarin injection. Histological evaluation showed that TP administration could inhibit pannus formation observed in PBS (−)administered mice (Figure 4b). The edema ratios of the joint and top of the forefoot and of the back foot in the TP-treated group were lower than those in the control group (Figure 5). Furthermore, the arthritis scores of the forefoot and back foot in the TP-treated group were lower than those in the control group (Figure 6). On quantifying plasma TNF-α levels using ELISA (LBIS Mouse TNF-α ELISA Kit, FUJIFILM Wako Shibayagi Corporation, Gunma, Japan), this level was lower in the TP-treated group than that in the control group; however, no statistical differences were observed (Figure 7a). These results suggest that the TP-induced anti-arthritis effect might involve the decrease in plasma TNF-α level. During the experiments, the body weights in the TP-treated group were almost the same as those in the control group (Figure 7b). These results suggest that TP safely inhibited arthritis in RA model mice.

In the present study, the in vivo anti-RA effects of TP were indicated by the TP-mediated inhibition of RA-related gene expression in HFLS-RA cells. However, TP might act as a provitamin of α-T because TP is reportedly converted to α-T in vivo [46]. Therefore, the in vivo anti-RA effects of α-T itself need to be determined in future investigations.

## 3. Materials and Methods

### 3.1. Materials

α-T, TS, TP, 2,2,5,7,8-pentamethyl-6-chromanol (PMC), and laminarin from *Laminaria digitata* were purchased from Sigma-Aldrich (St. Louis, MO, USA). Human TNF-α protein was obtained from PeproTech, Inc. (Cranbury, NJ, USA). Premix WST-1 Cell Proliferation Assay System, Prime Script Reverse Transcriptase, and SYBR Premix Ex Taq II were obtained from Takara Bio Inc. (Shiga, Japan). Human fibroblast-like synoviocytes: Rheumatoid Arthritis (HFLS-RA) and Synoviocyte Growth Medium Kit were obtained from Cell Applications, Inc. (San Diego, CA, USA). RNeasy Mini Kit and RNase-Free DNase Set were obtained from Qiagen (Valencia, CA, USA). Oligo dT primers were purchased from Thermo Fisher Scientific Inc. (Waltham, MA, USA). SKG/Jcl mice were obtained from CLEA Japan, Inc. (Tokyo, Japan).

### 3.2. Evaluation of Cell Viability by WST-1 Assay

HFLS-RA cells were propagated in synoviocyte growth medium at 37 °C, 21% O_2_, and 5% CO_2_ under humidified conditions. The cell viability assay was performed as described previously [47]. The cells were seeded on 96-well Cell BIND plates (Corning) at a density of 3 × 10^3^ cells/well. After incubation at 37 °C for 24 h, the cells were treated with 20, 50, and 100 μM α-T, TS, and TP for 24 h. Cell viability was determined by WST-1 assay according to the manufacturer’s instructions. Cell viability was estimated by dividing the absorbance (at 450 nm) of the sample by that of the untreated group.

### 3.3. Determination of mRNA Levels by RT-PCR

HFLS-RA cells were seeded on a 60 mm dish at a density of 2.5 × 10^5^ cells/dish. After incubation at 37 °C for 24 h, the cells were treated with 50 μM α-T, 20 μM TS, and 50 μM TP for 24 h. To stimulate HFLS-RA cells, HFLS-RA cells were treated with 20 ng/mL TNF-α. After cell collection, total RNA extraction and RT-PCR were performed as previously described [48]. Total RNA was isolated using the RNeasy Mini Kit and RNase-Free DNase Set, according to the manufacturer’s instructions. cDNA was synthesized using oligo dT primers and prime script reverse transcriptase. Real-time PCR was then performed by ABI PRISM 7500 Sequence Detection System (Applied Biosystems, Foster City, CA, USA) using SYBR Premix Ex Taq Ⅱ with the following specific primers: human IL-6 (forward: 5′-CAGCCACTCACCTCTTCAGAA-3′, reverse: 5′-GCTGCTTTCACACATGTTACTCTT-3′), human TNF-α (forward: 5′- TCAGAGGGCCTGTACCTCATCT-3′, reverse: 5′-TGTGGGTGAGGAGCACATG-3′), human MMP3 (forward: 5′-GGCACAATATGGGCACTTTA-3′, reverse: CCGGCAAGATACAGATTCAC-3′), human MMP13 (forward: 5′-AGTGGTGGTGATGAAGATGATTTG-3′, reverse: 5′-TCTCAGGTAGCGCTCTGCAA-3′) and human GAPDH (5′-GCACCGTCAAGGCTGAGAAC-3′, reverse: ATGGTGGTGAAGACGCCAGT-3′). Relative mRNA levels were determined by the 2^−∆∆Ct^ method using GAPDH mRNA as an internal control.

### 3.4. Quantification of TP Amounts in the Cell Culture Medium by HPLC

HFLS-RA cells were seeded in 12 well plate at a density of 5 × 10^4^ cells/well. After incubation at 37 °C for 24 h, the cells were treated with 50 μM TP for 24 h. The medium was collected and centrifuged at 2400× *g* for 2 min. The supernatant was mixed with 500 µM PMC as an internal standard substance, 0.1% trifluoroacetic acid (TFA) diluted in water, and 0.1% TFA diluted in methanol at a ratio of 10:1:10:79. After filtration through a 0.45 µm Millex-LH PTFE membrane filter (Merck Millipore, Darmstadt, Germany), the amount of TP in the sample was determined by HPLC-FL (JASCO EXTREMA Series, Tokyo, Japan) using a LUNA C8 (2) column 150 mm, 5 µm (Phenomenex, Torrance, CA, USA). In HPLC analysis, the flow rate, injection volume, and column temperature were 0.4 mL/min, 5 µL, and 40 °C, respectively. The 0.1% TFA in water (mobile phase A) and 0.1% TFA in methanol (mobile phase B) were controlled using the following gradient program: A 15% from 0 to 3 min, B 85–100% from 3 to 5 min, and B 100% from 5 to 12 min. The fluorescent signal of TP was detected at λ_ex_ = 285 nm and λ_em_ = 320 nm.

### 3.5. Animal Experiments

All mice were maintained and used in accordance with the animal protocol approved by the Institutional Animal Care and Use Committee of the Kyoto Pharmaceutical University (Kyoto, Japan). To prepare the mouse model of rheumatoid arthritis, 137 mg/mL laminarin dissolved in PBS (−) was intraperitoneally administered to SKG/Jcl mice at a dose of 30 mg/mouse [49]. A total of 7 days after the administration of laminarin, TP dissolved in PBS (−) was intraperitoneally administered to mice at a dose of 100 mg/kg, 3 times a week. In the control group, the same volume of PBS (−) was injected. The macroscopic findings of arthritis were assessed using arthritis scores [43]: 0, no change; 1, mild swelling; 2, clear swelling/reddening; 3, high swelling or edema; 4, degenerative joint. To evaluate edema in the four limbs, the length, width, and instep thickness were measured using calipers, and the ratio of edema was calculated using the following formula [50]: Edema ratio = {(volume after treatment) − (volume before treatment)}/(volume before treatment) × 100.

### 3.6. Statistical Analysis

Statistical significance was determined using Student’s *t*-test or one-way ANOVA, followed by Tukey’s honest significant test. Statistical significance was set at *p* < 0.05.

## 4. Conclusions

In the present study, we investigated the effects of α-T, TS, and TP on the expression of RA-related genes in FLS. Among these compounds, only TP inhibited the mRNA expression of IL-6, TNF-α, MMP-3, and MMP-13. Furthermore, TP showed anti-RA effects in a laminarin-induced RA mouse model. Therefore, TP is one of the natural forms of vitamin E with anti-RA effects and could be employed in RA therapy.

## Data Availability

The data presented in this study, are available in this article.

## Sample Availability

Samples of the compounds are not available from the authors.

## References

[1] Patidar V., Shah S., Kumar R., Singh P.K., Singh S.B., Khatri D.K. (2021). A molecular insight of inflammatory cascades in rheumatoid arthritis and anti-arthritic potential of phytoconstituents. Mol. Biol. Rep..

[2] Jones D.S., Jenney A.P., Swantek J.L., Burke J.M., Lauffenburger D.A., Sorger P.K. (2017). Profiling drugs for rheumatoid arthritis that inhibit synovial fibroblast activation. Nat. Chem. Biol..

[3] McInnes I.B., Leung B.P., Liew F.Y. (2000). Cell-cell interactions in synovitis: Interactions between T lymphocytes and synovial cells. Arthritis Res. Ther..

[4] Gaur U., Aggarwal B.B. (2003). Regulation of proliferation, survival and apoptosis by members of the TNF superfamily. Biochem. Pharmacol..

[5] Scott B.B., Weisbrot L.M., Greenwood J.D., Bogoch E.R., Paige C.J., Keystone E.C. (1997). Rheumatoid arthritis synovial fibroblast and U937 macrophage/monocyte cell line interaction in cartilage degradation. Arthritis Rheum..

[6] Liu N., Feng X., Wang W., Zhao X., Li X. (2017). Paeonol protects against TNF-α-induced proliferation and cytokine release of rheumatoid arthritis fibroblast-like synoviocytes by upregulating FOXO3 through inhibition of miR-155 expression. Inflamm. Res..

[7] Lee G.-H., Lee J., Lee J.-W., Choi W.S., Moon E.-Y. (2013). B cell activating factor-dependent expression of vascular endothelial growth factor in MH7A human synoviocytes stimulated with tumor necrosis factor-α. Int. Immunopharmacol..

[8] Yamana J., Morand E.F., Manabu T., Sunahori K., Takasugi K., Makino H., Yamamura M. (2012). Inhibition of TNF-induced IL-6 by the TWEAK-Fn14 interaction in rheumatoid arthritis fibroblast like synoviocytes. Cell. Immunol..

[9] Zhang C., Chang J., Wu W., Deng Y., Zhou P., Jiang W., Wang C., Huang F. (2020). Activation of GPR43 suppresses TNF-α-induced inflammatory response in human fibroblast-like synoviocytes. Arch. Biochem. Biophys..

[10] Sommerfelt R.M., Feuerherm A.J., Jones K., Johansen B. (2013). Cytosolic Phospholipase A2 Regulates TNF-Induced Production of Joint Destructive Effectors in Synoviocytes. PLoS ONE.

[11] Jiang F., Zhou H.Y., Zhou L.F., Zeng W., Zhao L.H. (2020). IRF9 affects the TNF-induced phenotype of rheumatoid-arthritis fibroblast-like synoviocytes via regulation of the SIRT-1/NF-kappaB signaling pathway. Cells Tissues Organs.

[12] Koedderitzsch K., Zezina E., Li L., Herrmann M., Biesemann N. (2021). TNF induces glycolytic shift in fibroblast like synoviocytes via GLUT1 and HIF1A. Sci. Rep..

[13] Xu D.W., Zhu X.H., He M.Q., Yuan Q., Dong Q.R. (2019). beta4GalT1 promotes inflammation in human osteoarthritic fibroblast-like synoviocytes by enhancing autocrine TNF-alpha activity. Eur. Rev. Med. Pharmacol. Sci..

[14] Inoue A., Matsumoto I., Tanaka Y., Umeda N., Takai C., Kawaguchi H., Ebe H., Yoshida H., Matsumoto Y., Segawa S. (2016). TIARP attenuates autoantibody-mediated arthritis via the suppression of neutrophil migration by reducing CXCL2/CXCR2 and IL-6 expression. Sci. Rep..

[15] Cheng W.X., Huang H., Chen J.H., Zhang T.T., Zhu G.Y., Zheng Z.T., Lin J.T., Hu Y.P., Zhang Y., Bai X.L. (2019). Genistein inhibits angiogenesis developed during rheumatoid arthritis through the IL-6/JAK2/STAT3/VEGF signalling pathway. J. Orthop. Transl..

[16] Kimura A., Kishimoto T. (2010). IL-6: Regulator of Treg/Th17 balance. Eur. J. Immunol..

[17] Wang T., He C. (2020). TNF-α and IL-6: The Link between Immune and Bone System. Curr. Drug Targets.

[18] Bek S., Bojesen A.B., Nielsen J.V., Sode J., Bank S., Vogel U., Andersen V. (2017). Systematic review and meta-analysis: Pharmacogenetics of anti-TNF treatment response in rheumatoid arthritis. Pharm. J..

[19] McInnes I.B., Schett G. (2017). Pathogenetic insights from the treatment of rheumatoid arthritis. Lancet.

[20] Kang S., Tanaka T., Narazaki M., Kishimoto T. (2019). Targeting Interleukin-6 Signaling in Clinic. Immunity.

[21] Ogata A., Kato Y., Higa S., Yoshizaki K. (2019). IL-6 inhibitor for the treatment of rheumatoid arthritis: A comprehensive review. Mod. Rheumatol..

[22] Wang W., Sun W., Jin L. (2017). Caffeic acid alleviates inflammatory response in rheumatoid arthritis fibroblast-like synoviocytes by inhibiting phosphorylation of IkappaB kinase alpha/beta and IkappaBalpha. Int. Immunopharmacol..

[23] Yang K.-C., Wu C.-C., Chen W.-Y., Sumi S., Huang T.-L. (2016). l-Glutathione enhances antioxidant capacity of hyaluronic acid and modulates expression of pro-inflammatory cytokines in human fibroblast-like synoviocytes. J. Biomed. Mater. Res. Part A.

[24] Kunsch C., Luchoomun J., Chen X.-L., Dodd G.L., Karu K.S., Meng C.Q., Marino E.M., Olliff L.K., Piper J.D., Qiu F.-H. (2005). AGIX-4207 [2-[4-[[1-[[3,5-Bis(1,1-dimethylethyl)-4-hydroxyphenyl]thio]-1-methylethyl]thio]-2,6-bis(1,1-dimethylethyl)phenoxy]acetic Acid], a Novel Antioxidant and Anti-Inflammatory Compound: Cellular and Biochemical Characterization of Antioxidant Activity and Inhibition of Redox-Sensitive Inflammatory Gene Expression. J. Pharmacol. Exp. Ther..

[25] Niki E., Traber M.G. (2012). A History of Vitamin E. Ann. Nutr. Metab..

[26] Niki E. (2014). Role of vitamin E as a lipid-soluble peroxyl radical scavenger: In vitro and in vivo evidence. Free Radic. Biol. Med..

[27] Edmonds S.E., Winyard P.G., Guo R., Kidd B., Merry P., Langrish-Smith A., Hansen C., Ramm S., Blake D.R. (1997). Putative analgesic activity of repeated oral doses of vitamin E in the treatment of rheumatoid arthritis. Results of a prospective placebo controlled double blind trial. Ann. Rheum. Dis..

[28] Kogure K., Hama S., Manabe S., Tokumura A., Fukuzawa K. (2002). High cytotoxicity of α-tocopheryl hemisuccinate to cancer cells is due to failure of their antioxidative defense systems. Cancer Lett..

[29] Hama S., Okamura Y., Kamei K., Nagao S., Hayashi M., Shizuka M., Fukuzawa K., Kogure K. (2019). α-Tocopheryl succinate stabilizes the structure of tumor vessels by inhibiting angiopoietin-2 expression. Biochem. Biophys. Res. Commun..

[30] Nakamura T., Goto M., Matsumoto A., Tanaka I. (1998). Inhibition of NF-kappa B transcriptional activity by alpha-tocopheryl succinate. Biofactors.

[31] Zingg J.-M. (2018). Water-Soluble Vitamin E—Tocopheryl Phosphate. Adv. Food Nutr. Res..

[32] Munteanu A., Zingg J.-M., Ogru E., Libinaki R., Gianello R., West S., Negis Y., Azzi A. (2004). Modulation of cell proliferation and gene expression by α-tocopheryl phosphates: Relevance to atherosclerosis and inflammation. Biochem. Biophys. Res. Commun..

[33] Ricciarelli R., Zingg J.-M., Azzi A. (2000). Vitamin E Reduces the Uptake of Oxidized LDL by Inhibiting CD36 Scavenger Receptor Expression in Cultured Aortic Smooth Muscle Cells. Circulation.

[34] Zingg J.M., Azzi A., Meydani M. (2015). Induction of VEGF expression by α-tocopherol and α-tocopheryl phosphate via PI3Kgamma/PKB and hTAP1/SEC14L2-mediated lipid exchange. J. Cell. Biochem..

[35] Zingg J.M., Azzi A., Meydani M. (2017). α-Tocopheryl phosphate induces VEGF expression via CD36/PI3Kgamma in THP-1 monocytes. J. Cell. Biochem..

[36] Crouzin N., Ferreira M.-C.D.J., Cohen-Solal C., M’Kadmi C., Bernad N., Martinez J., Barbanel G., Vignes M., Guiramand J. (2011). α-Tocopherol and α-tocopheryl phosphate interact with the cannabinoid system in the rodent hippocampus. Free Radic. Biol. Med..

[37] Nakayama S., Kobayashi S., Katoh E.M., Tsuzuki T. (2003). Protective Effect of α-Tocopherol-6-O-Phosphate against Ultraviolet B-Induced Damage in Cultured Mouse Skin. J. Investig. Dermatol..

[38] Libinaki R., Vinh A., Widdop R., Gaspari T., Tesanovic-Klajic S. (2017). The effect of tocopheryl phosphates (TPM) on the development of atherosclerosis in apolipoprotein-E deficient mice. Clin. Exp. Pharmacol. Physiol..

[39] Libinaki R., Tesanovic S., Heal A., Nikolovski B., Vinh A., Widdop R.E., Gaspari T.A., Devaraj S., Ogru E. (2010). Effect of tocopheryl phosphate on key biomarkers of inflammation: Implication in the reduction of atherosclerosis progression in a hypercholesterolaemic rabbit model. Clin. Exp. Pharmacol. Physiol..

[40] Galleron S., Borderie D., Ponteziere C., Lemarechal H., Jambou M., Roch-Arveiller M., Ekindjian O., Cals M. (1999). Reactive oxygen species induce apoptosis of synoviocytes in vitro. α-tocopherol provides no protection. Cell Biol. Int..

[41] Sun H., Luo Y., Meng L., Piao X., Wang Y., Wang J., Wang H., Zhang Y., Li J., Xu W. (2018). Cryptotanshinone induces reactive oxygen species-mediated apoptosis in human rheumatoid arthritis fibroblast-like synoviocytes. Int. J. Mol. Med..

[42] Kogure K., Morita M., Nakashima S., Hama S., Tokumura A., Fukuzawa K. (2001). Superoxide is responsible for apoptosis in rat vascular smooth muscle cells induced by α-tocopheryl hemisuccinate. Biochim. Biophys. Acta BBA Gen. Subj..

[43] Bashir M.R., Rezk B.M., van der Vijgh W.J.F., Bast A., Haenen G.R.M.M. (2007). Alpha-tocopheryl phosphate is a novel apoptotic agent. Front. Biosci..

[44] Miwa N., Saitoh Y., Yumoto A. (2009). α-tocopheryl phosphate suppresses tumor invasion concurrently with dynamic morphological changes and delocalization of cortactin from invadopodia. Int. J. Oncol..

[45] Negis Y., Meydani M., Zingg J.-M., Azzi A. (2007). Molecular mechanism of α-tocopheryl-phosphate transport across the cell membrane. Biochem. Biophys. Res. Commun..

[46] Nishio K., Ishida N., Saito Y., Ogawa-Akazawa Y., Shichiri M., Yoshida Y., Hagihara Y., Noguchi N., Chirico J., Atkinson J. (2011). α-Tocopheryl phosphate: Uptake, hydrolysis, and antioxidant action in cultured cells and mouse. Free Radic. Biol. Med..

[47] Hama S., Utsumi S., Fukuda Y., Nakayama K., Okamura Y., Tsuchiya H., Fukuzawa K., Harashima H., Kogure K. (2012). Development of a novel drug delivery system consisting of an antitumor agent tocopheryl succinate. J. Control. Release.

[48] Nakamura I., Hama S., Itakura S., Takasaki I., Nishi T., Tabuchi Y., Kogure K. (2014). Lipocalin2 as a plasma marker for tumors with hypoxic regions. Sci. Rep..

[49] Yoshitomi H., Sakaguchi N., Kobayashi K., Brown G.D., Tagami T., Sakihama T., Hirota K., Tanaka S., Nomura T., Miki I. (2005). A role for fungal {beta}-glucans and their receptor Dectin-1 in the induction of autoimmune arthritis in genetically susceptible mice. J. Exp. Med..

[50] Matsumoto N., Iinuma H., Sawa T., Takeuchi T., Hirano S.-I., Yoshioka T., Ishizuka M. (1997). Epoxyquinomicins A, B, C and D, New Antibiotics from Amycolatopsis. II. Effect on Type II Collagen-induced Arthritis in Mice. J. Antibiot..

